# A Case of Incidental Gastrointestinal Stromal Tumor in a Patient With Walled-Off Pancreatic Necrosis

**DOI:** 10.7759/cureus.10681

**Published:** 2020-09-27

**Authors:** Rahul Gupta, Arvind Singh, Houssem Ammar

**Affiliations:** 1 Gastroenterology, Synergy Institute of Medical Sciences, Dehradun, IND; 2 Surgery, Sahloul Hospital, Sousse, TUN

**Keywords:** gastrointestinal stromal tumor (gist), walled-off pancreatic necrosis, digestive surgery, cystogastrostomy, deep venous thrombosis

## Abstract

Acute pancreatitis is a common clinical disorder of the pancreas that can present with walled-abdominal pain and vomiting. We report a case of a 45-year-old man with alcohol-related acute pancreatitis who developed a large walled-off pancreatic necrosis. Incidentally, the patient had a small gastrointestinal stromal tumor in the adjoining wall of the stomach that was missed on preoperative radiological imaging. A small submucosal lesion was detected during cystogastrostomy and was excised. The diagnosis of a gastrointestinal stromal tumor was confirmed on histopathology.

## Introduction

Acute pancreatitis is a commonly encountered pancreatic disease in clinical practice. Most cases are mild but about 10%-20% of cases can develop severe acute pancreatitis. Severe acute pancreatitis is associated with significant morbidities and mortality. Walled-off pancreatic necrosis (WOPN) is a frequent sequela of severe acute pancreatitis. Most of these patients have persistent abdominal pain, fever, vomiting, or jaundice [1]. Mild anemia is often present in these patients due to nutritional deficiencies and anemia of chronic diseases. However, severe anemia in the absence of a bleeding pseudoaneurysm in the setting of WOPN is extremely rare. Secondly, portosplenic venous thrombosis is frequently observed with WOPN. Systemic venous complications, such as deep venous thrombosis (DVT) and pulmonary thromboembolism (PTE), are rare. In addition, WOPN can be misdiagnosed as a gastrointestinal stromal tumor (GIST) on radiology but the coexistence of both has not been reported in the literature [2]. We report a rare case of WOPN with incidental gastric GIST detected and excised during surgery.

## Case presentation

**Figure 1:**
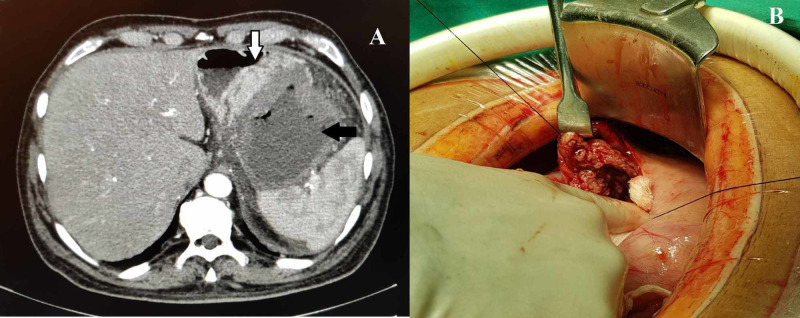
(A) Contrast-enhanced computed tomography of the abdomen showing the walled-off pancreatic necrosis with air foci within it (black arrow) and the hypervascular lesion in the gastric wall (white arrow). (B) Intraoperative photograph showing the gastric submucosal lesion (GIST).

A 45-year-old male presented to our emergency department with a two-day history of melena, fever, and abdominal pain. He had a past history of severe anemia (hemoglobin (Hb) - 5 gm/dl) requiring four units of blood transfusion three months back. At the same time, he was diagnosed with DVT for which he was taking oral anticoagulants. He was a chronic alcohol abuser consuming about one quarter of alcohol every day for 15 years. On clinical examination, the patient was pale, tachypneic, and had tachycardia with normal blood pressure. There was no calf tenderness or pedal edema. An ill-defined, non-tender lump was palpable in the epigastric and left hypochondriac region. Blood investigations revealed severe anemia and coagulopathy (Hb - 6.8 gm/dl, activated partial thromboplastin - 61 sec (normal range: 21-34 sec)). Contrast-enhanced computed tomography (CECT) of the abdomen revealed a well-defined, bilobed WOPN of 8 x 8 x 11 cm with multiple air foci within it. The part of WOPN abutting the gastric wall showed increased focal vascularity suspected to be due to collateral formation (Figure 1).

Additionally, hypodense filling defects were present in the inferior vena cava (IVC), bilateral common iliac veins, and left external iliac vein suggestive of thrombosis. Esophagogastroscopy found a large pulsatile bulge in the gastric fundus with a mild irregularity of the overlying mucosa. Since our center did not have an endoscopic ultrasound, and there was a risk of bleeding due to coagulopathy and extensive collaterals during endoscopic drainage without endoscopic ultrasound, surgery was planned.

On laparotomy, there was a large collection in the retrogastric space pushing the stomach anteriorly. After anterior gastrotomy, a 3 cm prominent bulge was present in the posterior wall of the stomach. The collection was punctured through the posterior gastric wall, and about 500 ml of foul-smelling pus was drained. While creating the cystogastrostomy opening, a 3 cm submucosal lesion was noted in the gastric wall (Figure 1). The lesion was excised along with the adjoining wall of the WOPN. A wide internal communication was created, and cystogastrostomy was performed. About 30 gm of necrosum was also removed. Hemostasis was achieved, and the anterior gastrotomy was closed. The operative time was 180 minutes with blood loss of 300 ml. In the postoperative period, the patient had worsening of the left lower limb swelling, which gradually subsided after restarting the anticoagulation.

**Figure 2:**
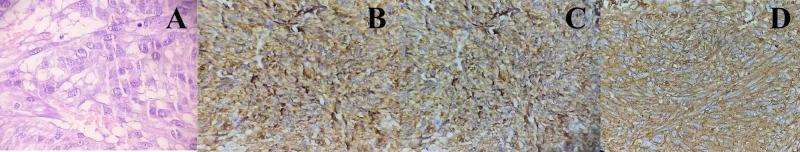
Microscopic examination showed spindle cells having fusiform nuclei and pale eosinophilic cytoplasm with scanty intervening stroma (H & E, 200x) (A); immunohistochemistry revealed strongly positive staining of the tumor cells with CD117 (B), CD 34 (C) and DOG1 (D) suggestive of GIST. GIST - gastrointestinal stromal tumor; H & E - hematoxylin and eosin; CD - cluster of differentiation; DOG1 - Discovered on GIST-1

Histopathological examination of the submucosal lesion revealed GIST arising from the stomach (Figure 2).

Imatinib therapy was not started due to the small size and complete resection of the lesion. At the last follow-up at 17 months, the patient was symptom-free and on oral anticoagulation.

## Discussion

GISTs are the most common mesenchymal tumors of the gastrointestinal tract, with the most common site being the stomach [3]. Rarely, GIST can coexist with other primary gastrointestinal tumors [4]. However, the incidental detection of GIST in the setting of WOPN has not been reported in the English literature to date. The index case presents an extremely rare coexistence of gastric GIST and WOPN. Moreover, previous studies have reported the misdiagnosis of gastric GIST as WOPN or pancreatic pseudocyst [2]. In the present case, although an area of hypervascularity was noted on CECT, GIST was not suspected preoperatively. We believe that a careful examination of the CECT abdomen is extremely important to avoid missing any other pathologies. In doubtful cases, endoscopic ultrasound can be done. Endoscopic ultrasound is highly sensitive and specific in detecting submucosal lesions of the stomach [5]. Recently, endoscopic drainage of the WOPN has become the preferred treatment due to lower complications as compared to surgery [6]. However, the presence of extensive peri-gastric collaterals or co-existent GIST, as seen in the present case, would be a relative contraindication for endoscopic treatment.

Another unique feature of the index case was the presence of extensive DVT. Venous thrombosis involving portosplenic circulation is frequently present in patients with pancreatic necrosis due to various factors such as mass effect from the inflamed pancreas, stasis, and increased levels of procoagulant inflammatory mediators [7]. Venous thrombosis involving IVC due to WOPN has been rarely reported [8]. The development of DVT in the setting of WOPN could be due to hypercoagulable state, compression of the IVC by pancreatic collection, or rupture of the pancreatic collection into the main venous channel. We believe that the present case suggests that pancreatic necrosis can not only lead to portosplenic venous complications but also systemic venous complications such as DVT, erosion of the adjoining systemic vessels, and PTE [8-9].

The index case had severe anemia on presentation mainly due to recurrent bleeding from the eroded gastric mucosa overlying the WOPN. However, there can be multiple contributory factors responsible for anemia in WOPN or acute necrotizing pancreatitis. These factors include bleeding pseudoaneurysms [10], hemorrhagic gastritis [11], co-existing hemolytic anemia, malarial or dengue fever [12], nutritional anemia, and sepsis [10]. Hence, appropriate evaluation and timely treatment is important in the prevention and treatment of anemia in such complicated cases.

The treatment of WOPN includes medical therapy with antibiotics, percutaneous drainage, endoscopic transgastric drainage, and surgery. Small WOPN (< 5 cm) resolves spontaneously with medical therapy. The preferred procedure for a large WOPN (> 5 cm) is endoscopic ultrasound-guided transgastric drainage. However, the presence of extensive collaterals, pseudoaneurysm, and hemodynamic instability are relative contraindications for endoscopic drainage. Moreover, endoscopic drainage requires advanced endoscopic equipment and expertise. Open or laparoscopic internal drainage is the treatment of choice in most cases. In the present case, due to coagulopathy, the presence of collaterals, and the lack of equipment, surgical drainage was performed.

## Conclusions

WOPN can lead to various uncommon complications such as anemia and extensive deep venous thrombosis. CECT or endoscopic ultrasound should be routinely performed and carefully studied to rule out the presence of any incidental co-existent submucosal lesions, such as GIST, before planning the treatment strategy.
